# Supplementary material for: Identification of novel allosteric sites of SARS-CoV-2 papain-like protease (PLpro) for the development of COVID-19 antivirals
Source: J Biol Chem. 2024 Sep 27;300(11):107821. doi: 10.1016/j.jbc.2024.107821 (PMC11538808; doi:10.1016/j.jbc.2024.107821)
Supplement: Supporting Information [file mmc1.pdf]

Supporting Information

Identification of Novel Allosteric Sites of SARS-CoV-2 Papain-Like Protease (PLpro) for the Development of COVID-19 Antivirals

Juliana C. Ferreira[1], Adrian J. Villanueva[1], Kenana Al Adem[1], Samar Fadl[1], Lara Alzyoud[2,3], Mohammad A Ghattas[2,3] and Wael M. Rabeh[1,*]

[1]Science Division, New York University Abu Dhabi, PO Box 129188, Abu Dhabi, United Arab Emirates.
[2]College of Pharmacy, Al Ain University, Abu Dhabi 64141, United Arab Emirates
[3]AAU Health and Biomedical Research Center, Al Ain University, Abu Dhabi 64141, United Arab Emirates

[*]Corresponding author: wael.rabeh@nyu.edu

Keywords: COVID-19, SARS-CoV-2, papain-like protease (PLpro), allosteric sites, thermodynamic stability, initial velocity studies.

Table S1. Mean values of the druggability score (*D*score) and other SiteMap physiochemical parameters obtained from the 22 PLpro structures analyzed in this work.

Site	*D*score	Size	Enclosure	Hydrophilicity
**Active Site**	0.88	75	0.68	0.99
**Pocket 1**	0.75	61	0.67	1.20
**Pocket 2**	0.57	29	0.58	0.89
**Pocket 3**	0.57	31	0.62	0.99

Table S2. Residues of the computationally predicted druggable pockets of SARS-CoV-2 PLpro. Only amino acids with potentially important side chain interactions were selected for further mutagenesis and biochemical analyses.

Site	Residues
**Pocket 1**	D12, N13, I14, L36, D37, G38, K53, T54, F55, Y56, Y71, Y72, Y83, L87, K91, A131, D134, A135, R138, E143, A144, A145, N146, L150
**Pocket 2**	L120, Q122, I123, E124, L125, K126, F127, Q133, Y136, Y137, R140, N177, L178, S180, C181, E238, S239, P240, F241, G256, F258, T259, T277, S278, K279
**Pocket 3**	S212, S213, E214, K217, Y251, E252, L253, K254, T257, F258, V303, Y305, K306, E307

Table S3. Kinetic parameters of Pocket 1 mutants of PLpro.

Variant	k_{cat} (min^{-1})<br>Fold Change	K_M (μM)<br>Fold Change	k_{cat}/K_M (min^{-1} • μM^{-1})<br>Fold Change
WT	4.75 ± 0.08	231 ± 6	0.0206 ± 0.0003
D12A	0.44 ± 0.01<br>**- 10.8**	273 ± 2<br>**+ 1.2**	0.0016 ± 0.0001<br>**- 12.8**
D12E	0.81 ± 0.03<br>**-5.9**	400 ± 10<br>**+ 1.7**	0.0020 ± 0.0001<br>**- 10.2**
D12N	2.80 ± 0.07<br>**-1.7**	420 ± 40<br>**+ 1.8**	0.0067 ± 0.0003<br>**- 3.1**
H17A	2.80 ± 0.10<br>**-1.7**	200 ± 10<br>**- 1.2**	0.0140 ± 0.0002<br>**- 1.5**
Y56A	1.70 ± 0.20<br>**-2.8**	300 ± 20<br>**+ 1.3**	0.0058 ± 0.0003<br>**- 3.6**
Y56F	2.77 ± 0.07<br>**- 1.7**	400 ± 10<br>**+ 1.7**	0.0070 ± 0.0001<br>**-2.9**
Y56T	2.10 ± 0.20<br>**- 2.3**	470 ± 40<br>**+ 2.0**	0.0045 ± 0.0003<br>**- 4.6**
E67A	0.70 ± 0.06<br>**- 6.8**	290 ± 20<br>**+ 1.3**	0.0024 ± 0.0001<br>**- 8.6**
Y71A	2.29 ± 0.05<br>**- 2.1**	137 ± 3<br>**- 1.7**	0.0167 ± 0.0001<br>**- 1.2**
Y71F	2.90 ± 0.70<br>**- 1.6**	700 ± 80<br>**+ 3.0**	0.0042 ± 0.0007<br>**- 4.9**
Y71T	1.90 ± 0.10<br>**- 2.5**	460 ± 30<br>**+ 2.0**	0.0042 ± 0.0002<br>**- 4.9**
Y72F	2.30 ± 0.07<br>**- 2.1**	420 ± 60<br>**+ 1.8**	0.0056 ± 0.0006<br>**- 3.7**
Y72T	0.22 ± 0.01<br>**- 22.0**	420 ± 30<br>**+ 1.8**	0.0005 ± 0.0001<br>**- 40.2**
Y83A	0.55 ± 0.03<br>**- 8.6**	206 ± 5<br>**- 1.1**	0.0027 ± 0.0001<br>**- 7.7**
Y83F	2.20 ± 0.10<br>**- 2.2**	460 ± 40<br>**+ 2.0**	0.0047 ± 0.0004<br>**- 4.3**
Y83T	0.25 ± 0.04<br>**-19**	440 ± 50<br>**+1.9**	0.0006 ± 0.0001<br>**-37.3**
K91A	3.70 ± 0.30<br>**- 1.3**	210 ± 20<br>**- 1.1**	0.0175 ± 0.0006<br>**- 1.2**
D134A	4.83 ± 0.05<br>**+ 1.0**	267 ± 7<br>**+ 1.2**	0.0181 ± 0.0003<br>**-1.1**
R138A	5.20 ± 0.60<br>**+ 1.1**	230 ± 30<br>**+ 1.0**	0.0221 ± 0.0008<br>**+ 1.1**
E143A	5.20 ± 0.08<br>**+ 1.1**	210 ± 8<br>**- 1.1**	0.0248 ± 0.0006<br>**+ 1.2**
N146A	6.40 ± 0.20<br>**+ 1.3**	240 ± 10<br>**+ 1.0**	0.0268 ± 0.0005<br>**+ 1.3**

Table S4. Kinetic parameters of Pocket 2 mutants of PLpro.

**Variant**	k_{cat} (min^{-1})<br>Fold Change	K_M (µM)<br>Fold Change	k_{cat}/K_M (min^{-1} • µM^{-1})<br>Fold Change
WT	4.75 ± 0.08	231 ± 6	0.0206 ± 0.0003
T119A	6.6 ± 0.4<br>**+ 1.4**	350 ± 50<br>**+ 1.5**	0.0198 ± 0.0004<br>**- 1.0**
Q122A	1.70 ± 0.20<br>**- 2.9**	190 ± 30<br>**- 1.2**	0.0088 ± 0.0002<br>**- 2.4**
Q122E	1.21 ± 0.08<br>**- 3.9**	470 ± 10<br>**+ 2.1**	0.0026 ± 0.0002<br>**- 8.0**
Q133A	3.20 ± 0.10<br>**- 1.5**	177 ± 8<br>**- 1.3**	0.0179 ± 0.0009<br>**- 1.2**
Q133N	1.50 ± 0.10<br>**- 3.3**	470 ± 60<br>**+ 2.1**	0.0031 ± 0.0001<br>**- 6.6**
R140A	2.52 ± 0.05<br>**- 1.9**	220 ± 4<br>**- 1.1**	0.0115 ± 0.0001<br>**- 1.8**
N177A	3.23 ± 0.02<br>**- 1.5**	270 ± 10<br>**+ 1.2**	0.0118 ± 0.0004<br>**- 1.8**
D179A	4.90 ± 0.50<br>**+ 1.0**	470 ± 60<br>**+ 2.1**	0.0103 ± 0.0003<br>**- 2.0**
Q236A	1.04 ± 0.01<br>**- 4.6**	330 ± 3<br>**+ 1.4**	0.0032 ± 0.0001<br>**- 6.5**
E238A	2.90 ± 0.10<br>**- 1.6**	370 ± 40<br>**+ 1.6**	0.0081 ± 0.0005<br>**- 2.5**
E238D	2.00 ± 0.20<br>**- 2.4**	410 ± 20<br>**+ 1.8**	0.0049 ± 0.0003<br>**- 4.2**
E238Q	2.20 ± 0.10<br>**- 2.2**	420 ± 40<br>**+ 1.8**	0.0053 ± 0.0005<br>**- 3.9**
S239A	0.15 ± 0.01<br>**- 32.6**	330 ± 30<br>**+ 1.4**	0.0004 ± 0.0001<br>**- 46.9**
H255A	1.12 ± 0.02<br>**- 4.3**	301<br>± 1<br>**+ 1.3**	0.0037 ± 0.0001<br>**- 5.5**
T277A	1.53 ± 0.06<br>**- 3.1**	150 ± 10<br>**- 1.6**	0.0106 ± 0.0004<br>**- 1.9**
S278A	0.89 ± 0.05<br>**- 5.3**	157 ± 6<br>**- 1.5**	0.0057 ± 0.0002<br>**- 3.6**
S278T	0.17 ± 0.01<br>**- 28.7**	500 ± 30<br>**+ 2.2**	0.0003 ± 0.00002<br>**- 62.7**
K279A	4.00 ± 0.20<br>**- 1.2**	240 ± 10<br>**+ 1.0**	0.0171 ± 0.0003<br>**- 1.2**

Table S5. Kinetic parameters of Pocket 3 mutants of PLpro.

**Variant**	k_{cat} (min^{-1})<br>Fold Change	K_M (µM)<br>Fold Change	k_{cat}/K_M (min^{-1} • µM^{-1})<br>Fold Change
WT	4.75 ± 0.08	231 ± 6	0.0206 ± 0.0003
S212A	0.50 ± 0.03<br>**- 9.6**	310 ± 30<br>**+ 1.3**	0.0016 ± 0.0001<br>**- 12.7**
S212T	0.04 ± 0.01<br>**- 126.3**	600 ± 100<br>**+ 2.8**	0.00006 ± 0.00001<br>**- 343.5**
Y213F	0.90 ± 0.10<br>**- 5.5**	430 ± 80<br>**+ 1.9**	0.0020 ± 0.0001<br>**- 10.2**
E214A	2.80 ± 0.20<br>**- 1.7**	430 ± 30<br>**+ 1.9**	0.0064 ± 0.0001<br>**- 3.2**
K217A	0.13 ± 0.01<br>**- 36.5**	440 ± 20<br>**+ 1.9**	0.000295 ± 0.0001<br>**- 69.7**
Y251A	0.15 ± 0.01<br>**- 31.9**	310 ± 30<br>**+ 1.3**	0.0005 ± 0.0001<br>**- 42.4**
Y251F	2.80 ± 0.50<br>**- 1.7**	380 ± 70<br>**+ 1.6**	0.0074 ± 0.0007<br>**- 2.8**
Y251T	2.37 ± 0.01<br>**- 2.0**	330 ± 20<br>**+ 1.4**	0.0073 ± 0.0004<br>**- 2.8**
E252A	3.66 ± 0.09<br>**- 1.3**	273 ± 2<br>**+ 1.2**	0.0134 ± 0.0002<br>**- 1.5**
K254A	0.44 ± 0.01<br>**- 10.8**	224 ± 9<br>**- 1.0**	0.0020 ± 0.0001<br>**- 10.5**
T259A	1.17 ± 0.01<br>**- 4.0**	289 ± 3<br>**+ 1.3**	0.0040 ± 0.0001<br>**- 5.0**
Y305F	0.24 ± 0.03<br>**- 19.6**	800 ± 100<br>**+ 3.3**	0.0003 ± 0.00002<br>**- 64.3**
Y305T	0.016 ± 0.002<br>**- 297.1**	600 ± 100<br>**+ 2.6**	0.00003 ± 0.000003<br>**- 755.6**
K306A	2.60 ± 0.20<br>**- 1.9**	210 ± 20<br>**- 1.1**	0.0121 ± 0.0001<br>**- 1.7**
K306R	1.45 ± 0.04<br>**- 3.3**	453 ± 8<br>**+ 2.0**	0.00312 ± 0.0001<br>**- 6.4**
E307A	1.17 ± 0.02<br>**- 4.1**	289 ± 3<br>**+ 1.3**	0.0041 ± 0.0001<br>**- 5.1**

Table S6. Thermodynamic parameters of PLpro variants determined by DSC.

Pocket	Variant	T_{m1} (°C) Fold Change	T_{m2} (°C) Fold Change	ΔH_{cal} (kJ/mol) Fold Change
	WT	44.4 ± 0.4	51.9 ± 0.1	1094 ± 73
Pocket 1	T10A	49.1 ± 0.5 **+1.1**	55.5 ± 0.2 **+1.07**	684 ± 131 **-1.60**
Pocket 1	D12A	53.0 ± 0.3 **+ 1.2**	57.2 ± 0.1 **+ 1.10**	348 ± 18 **- 3.15**
Pocket 1	T54A	47.4 ± 1.5 **+1.1**	53.0 ± 1.0 **+1.02**	604 ± 47 **-1.81**
Pocket 1	Y72A	47.3 ± 0.4 **+1.1**	55.9 ± 0.1 **+1.1**	460 ± 35 **-2.4**
Pocket 1	Y83A	50.7 ± 0.2 **+ 1.1**	56.6 ± 0.1 **+ 1.1**	758 ± 35 **- 1.4**
Pocket 2	Q122A	45.6 ± 0.9 **+ 1.0**	55.3 ± 0.5 **+ 1.1**	723 ± 25 **+ 1.1**
Pocket 2	Q237A	50.9 ± 0.5 **+1.2**	57.7 ± 0.4 **+1.1**	694 ± 52 **-1.6**
Pocket 2	E238A	46.1 ± 0.4 **+ 1.0**	51.7 ± 0.2 **+ 1.0**	867 ±34 **- 1.3**
Pocket 2	S239A	50.7 ± 0.2 **+ 1.1**	55.8 ± 0.3 **+ 1.1**	1195 ± 103 **+ 1.1**
Pocket 2	H275A	48.3 ± 1.6 **+1.1**	56.2 ± 0.8 **+1.1**	717 ± 36 **-1.5**
Pocket 2	T277A	46.1 ± 0.3 **+ 1.0**	51.9 ± 0.1 **+ 1.0**	2749 ± 146 **+ 2.5**
Pocket 2	S278A	43.7 ± 0.2 **- 1.0**	53.2 ± 0.1 **+ 1.0**	618 ± 49 **- 1.8**
Pocket 3	S212A	48.5 ± 0.5 **+ 1.1**	56.2 ± 0.8 **+ 1.1**	879 ± 83 **- 1.2**
Pocket 3	Y213A	45.2 ± 0.2 **+ 1.0**	56.5 ± 0.2 **+ 1.1**	1854 ± 30 **+ 1.7**
Pocket 3	E214A	49.6 ± 0.5 **+1.1**	54.2 ± 0.3 **+1.0**	907 ± 25 **-1.2**
Pocket 3	K217A	47.0 ± 0.2 **+1.1**	53.7 ± 0.2 **+1.0**	588 ± 59 **-1.9**
Pocket 3	Y251A	49.9 ± 0.8 **+1.1**	57.0 ± 0.6 **+1.1**	823 ± 74 **-1.3**
Pocket 3	K254A	46.1 ± 0.7 **+ 1.0**	50.5 ± 0.7 **- 1.0**	597 ± 51 **- 1.8**
Pocket 3	T259A	50.2 ± 0.4 **+ 1.1**	56.7 ± 0.5 **+ 1.1**	921 ± 59 **- 1.2**
Pocket 3	Y305A	50.5 ± 0.3 **+ 1.1**	56.6 ± 0.3 **+ 1.1**	778 ± 54 **- 1.4**

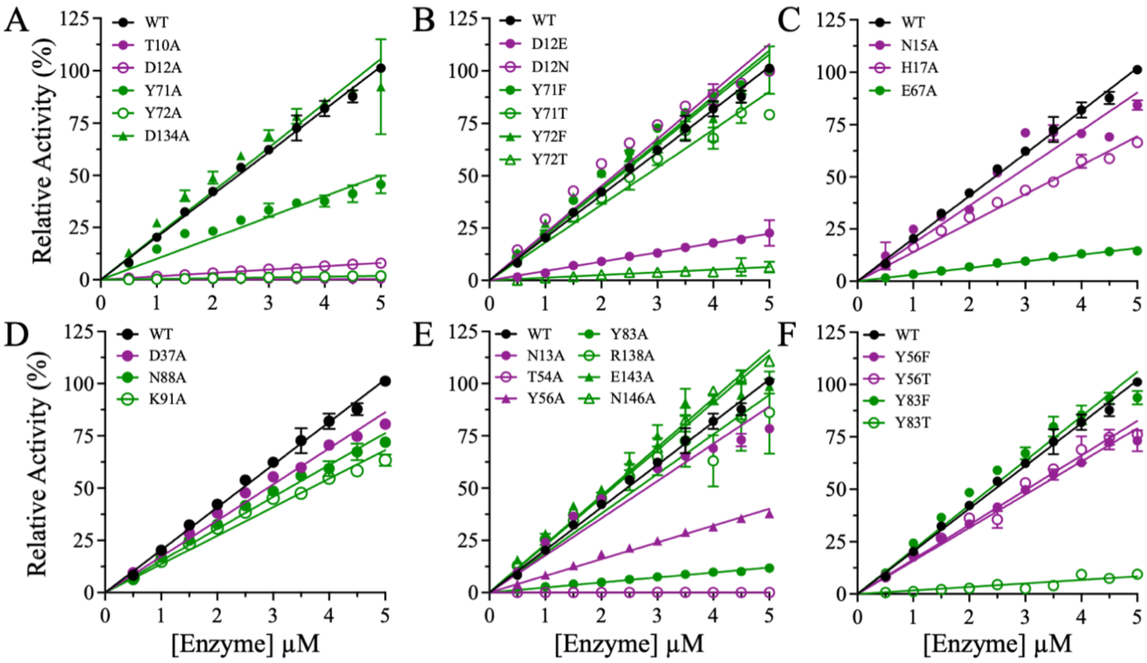

Figure S1. Relative activities of Pocket 1 mutants of PLpro. Activity was measured at increasing enzyme concentrations from 0.5 μM to 5.0 μM and a fixed peptide substrate concentration of 200 μM at 25 °C in buffer containing 20 mM HEPES pH 7.5, 150 mM NaCl, 1 mM EDTA, 1 mM TCEP, and 2% DMSO (v/v). The relative activities of the alanine mutants were obtained by normalizing the slopes of the lines to the slope of the line for WT PLpro. WT activity is shown as black-filled circles. The colors of the alanine mutants correspond to their domains: green = thumb and purple = Ubl. The data are presented as the mean ± SD, n = 3.

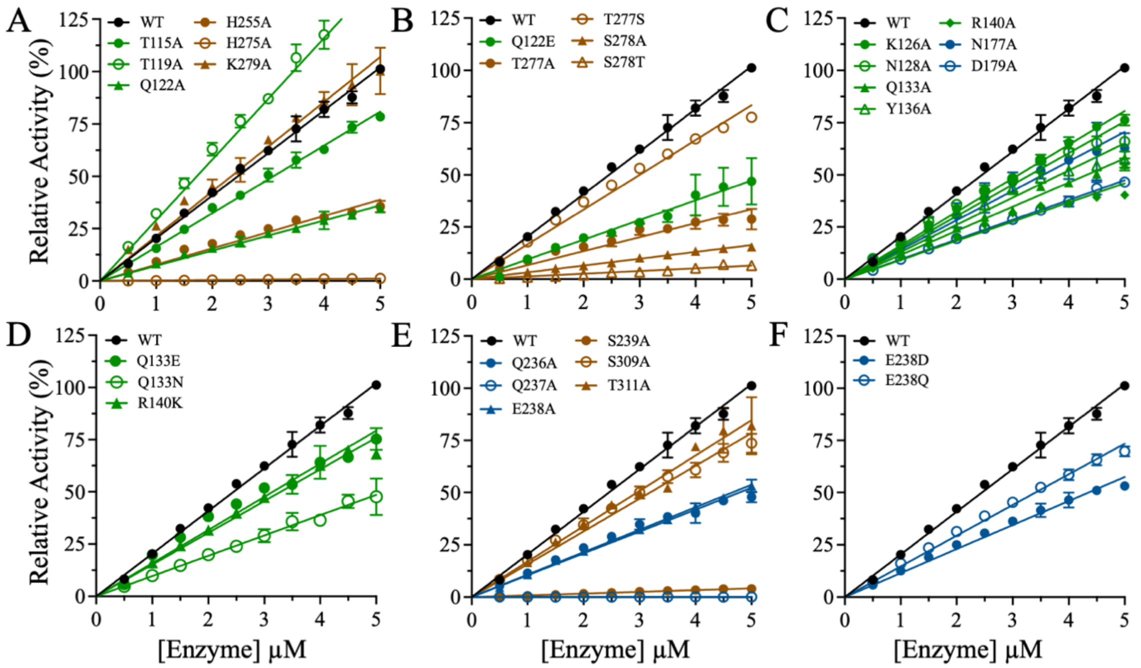

Figure S2: Relative activities of Pocket 2 mutants of PLpro. Relative activity was measured as described in Figure S1. WT activity is shown by black-filled circles. The colors of the alanine mutants correspond to their domains: green = thumb, blue = fingers, and brown = palm. The data are presented as the mean ± SD, n = 3.

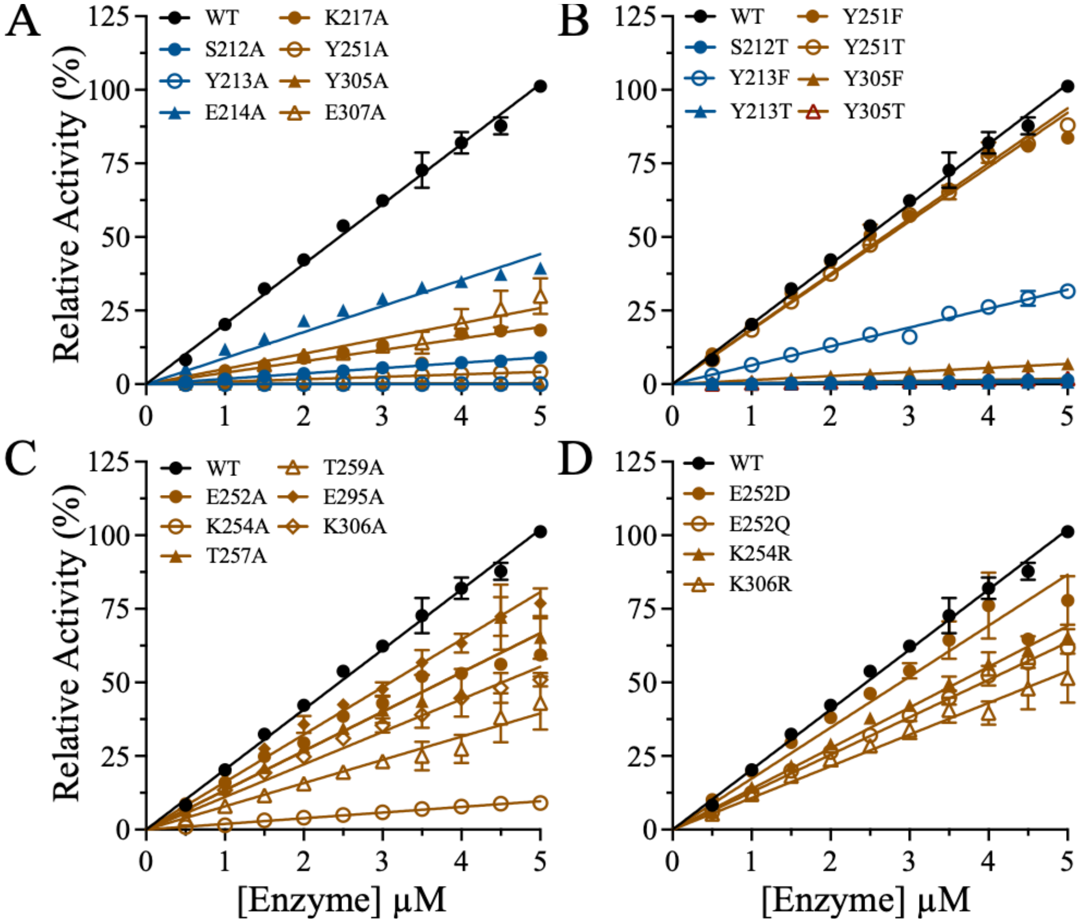

Figure S3: The relative activities of Pocket 3 mutants of PLpro. Relative enzymatic activity was measured as described in Figure S1. WT activity is shown by black-filled circles. The colors of the alanine mutants correspond to their domains: blue = fingers and brown = palm. The data are presented as the mean ± SD, n = 3.

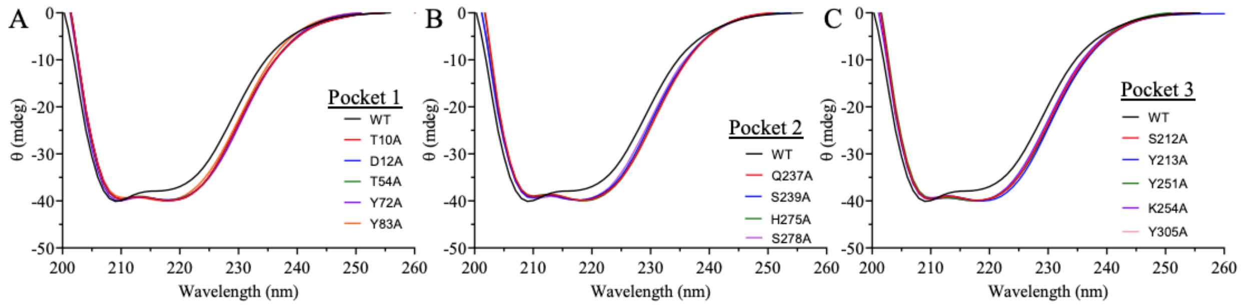

Figure S4: Circular dichroism (CD) scans. Far-UV CD scans of PLpro WT and variants were collected from 200 – 260 nm at 25 °C. All proteins, including WT and mutants PLpro in the three pockets investigated here, exhibited similar far-UV CD spectra with dual ellipticity minima at 208 nm and 222 nm. The spectrum of each protein is the average of five independent measurements. The CD scans of mutants in pockets 1, 2, and 3 are compared to the WT PLpro enzyme in panels A, B, and C, respectively.

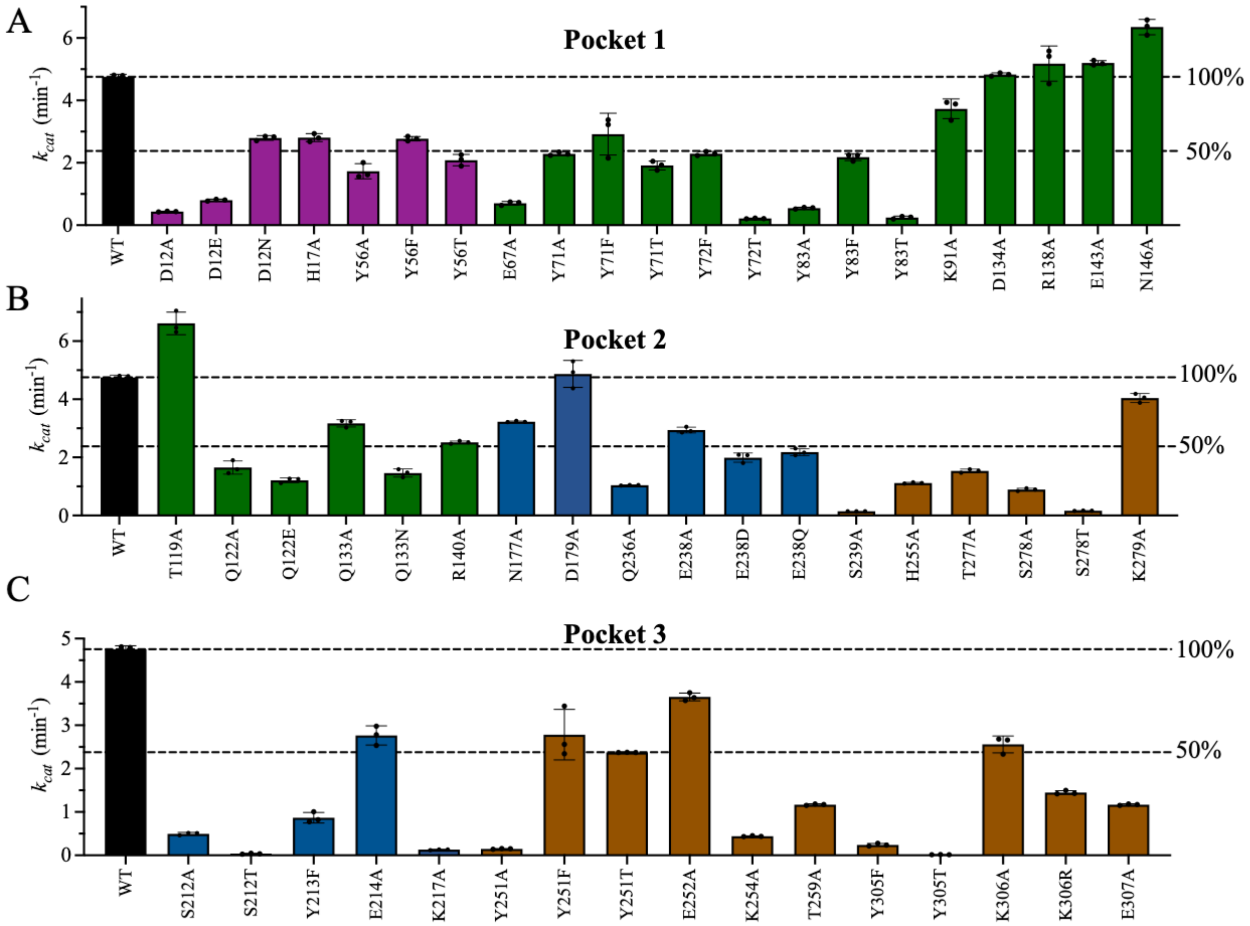

Figure S5: k_{cat} values of the enzymatically active PLpro mutants. Bar plots of the k_{cat} values of WT PLpro (black bars) and **(A)** Pocket 1 mutants, **(B)** Pocket 2 mutants, and **(C)** Pocket 3 mutants. The initial velocities were measured at 25 °C in buffer containing 20 mM HEPES pH 7.5, 150 mM NaCl, 1 mM EDTA, 1 mM TCEP, and 2% DMSO (v/v). The colors of the alanine mutants correspond to their domains: purple = Ubl, green = thumb, blue = fingers, and brown = palm. The bars represent the mean ± SD, n=3.

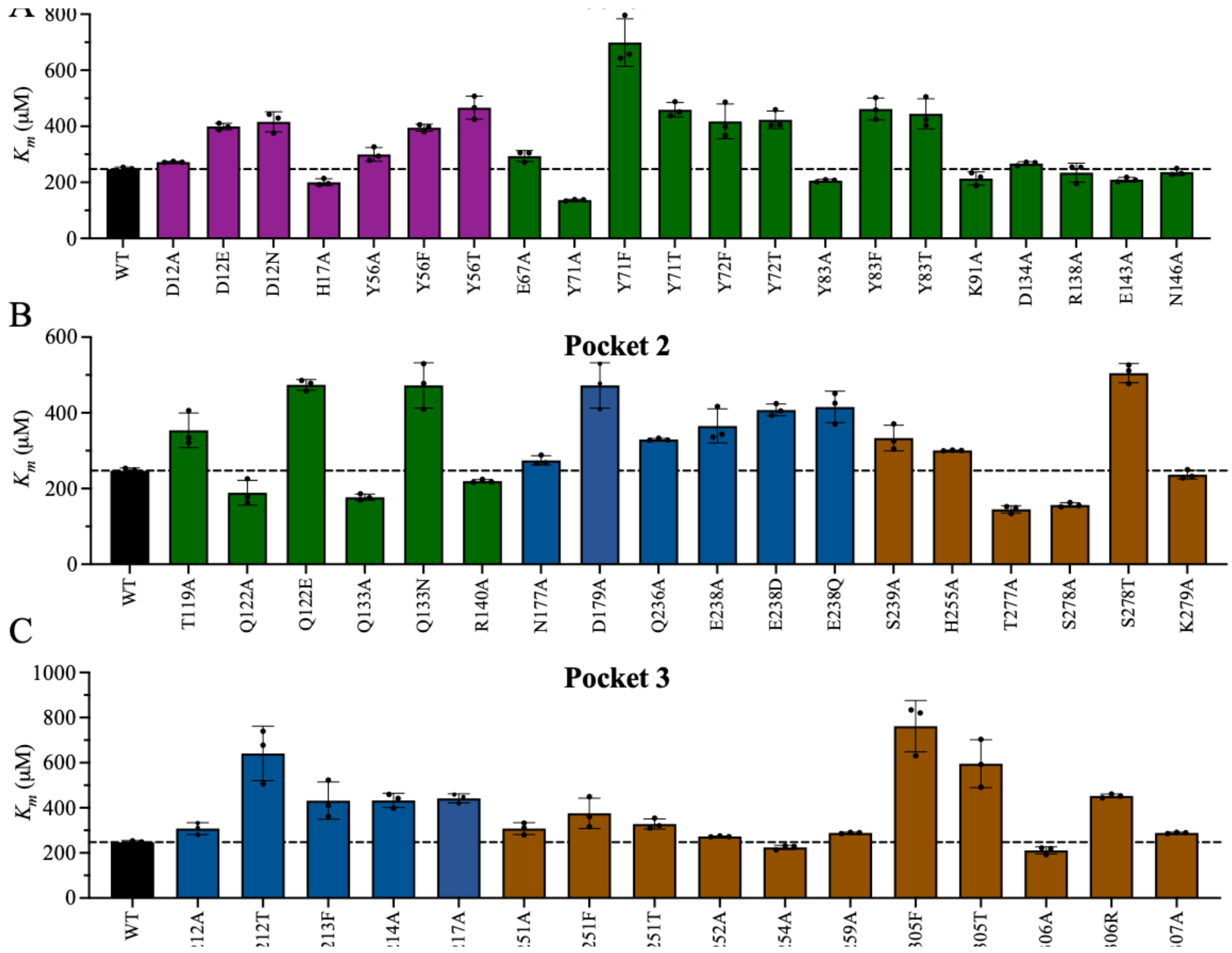

Figure S6: K_M values of the enzymatically active PLpro mutants. Bar plots of the K_m values of WT PLpro (black bars) and **(A)** Pocket 1 mutants, **(B)** Pocket 2 mutants, and **(C)** Pocket 3 mutants. The initial velocities were measured as described in Figure S4. The colors of the alanine mutants correspond to their domains: purple = Ubl, green = thumb, blue = fingers, and brown = palm. The bars represent the mean ± SD, n=3.

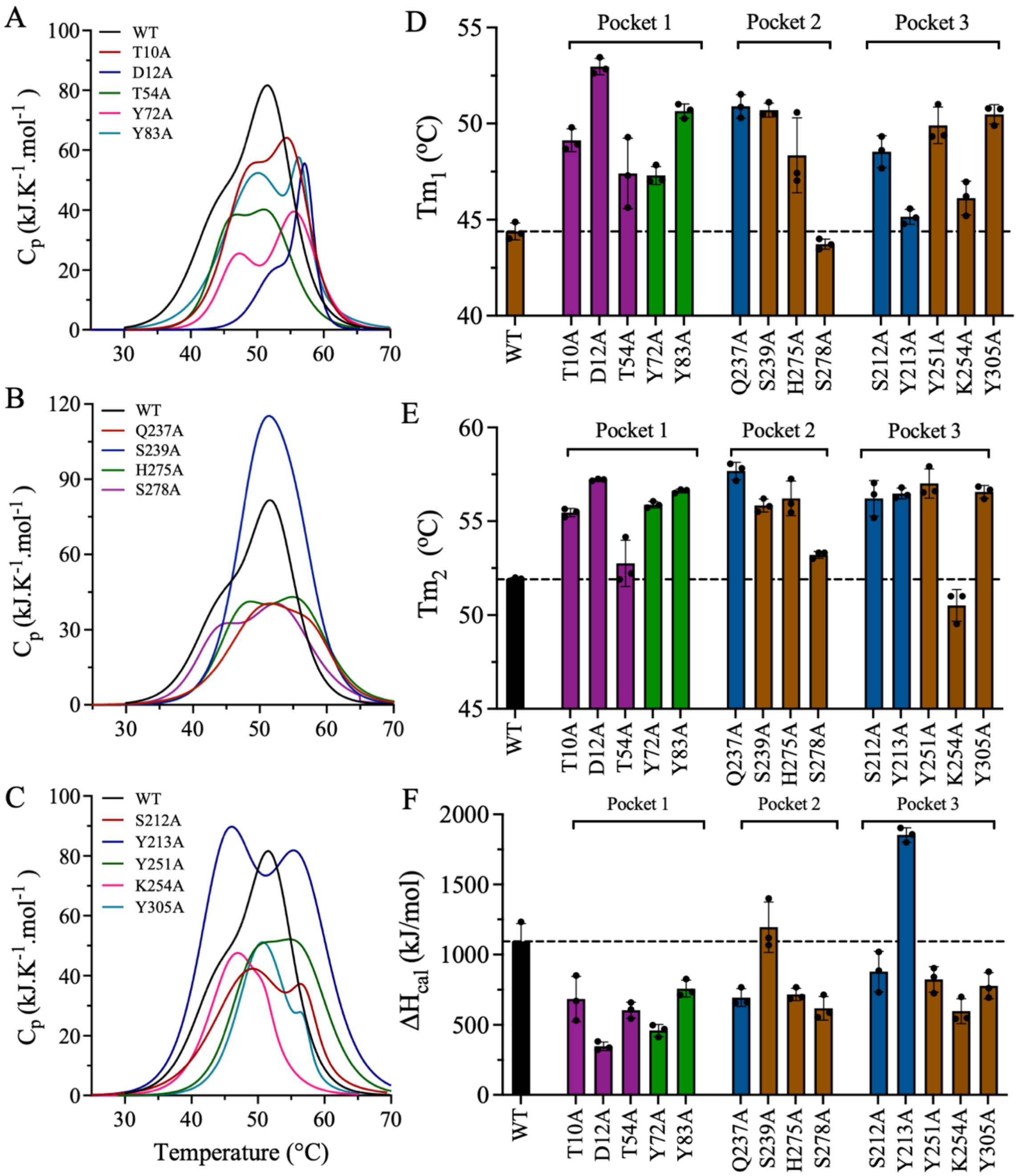

Figure S7. Thermodynamic parameters of WT PLpro and inactive mutants of Pockets 1–3. (A-C) DSC thermograms of the inactive mutants of **(A)** Pocket 1, **(B)** Pocket 2, and **(C)** Pocket 3. The thermograms were deconvoluted to a two-state transition with two T_m values calculated at the two apexes of the thermographic peaks. **(F)** Bar plots of T_{m1} and **(G)** T_{m2} of WT PLpro and the inactive mutants of Pockets 1–3. **(H)** Bar plots of the ΔH_{cal} values of WT PLpro and the inactive mutants of Pockets 1–3 calculated from the area under the thermographic peak. Data are the mean ± SD, n=3.

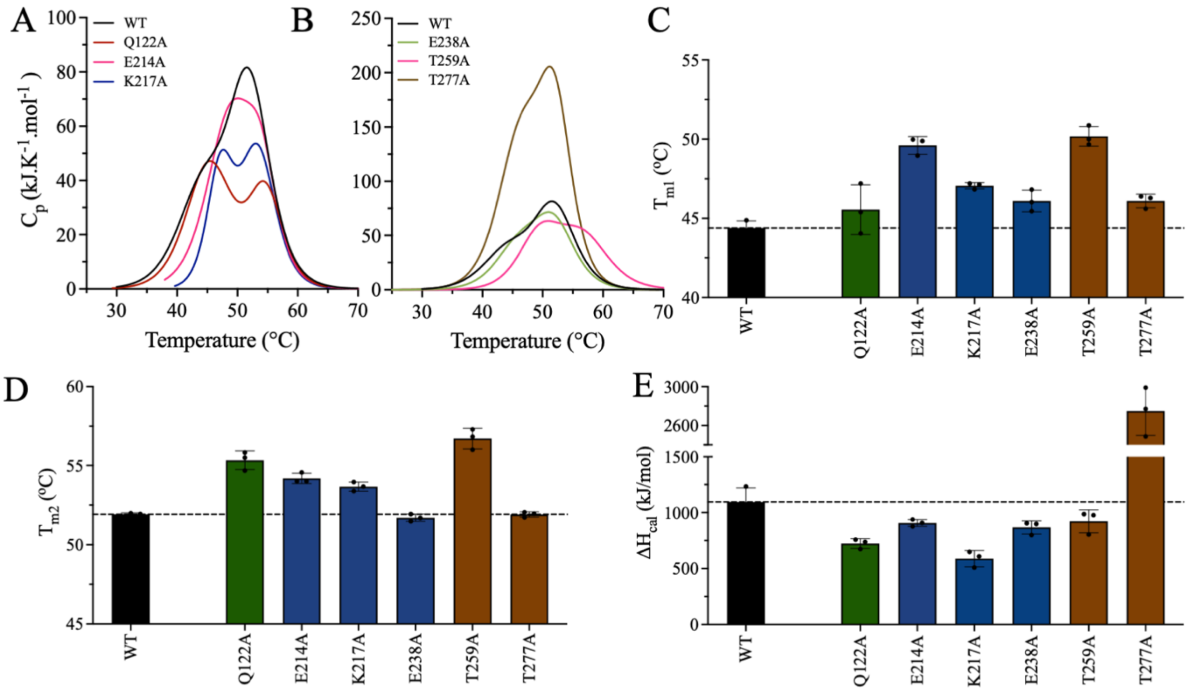

Figure S8. Thermodynamic parameters of WT PLpro and partially active mutants of Pockets 1–3. (A–B) DSC thermograms of the partially active mutants of Pockets 1–3. **(C)** Bar plots of T_{m1}, **(D)** T_{m2}, and **(E)** the ΔH_{cal} values of WT PLpro and the partially active mutants of Pockets 1–3. Data are the mean ± SD, n=3.

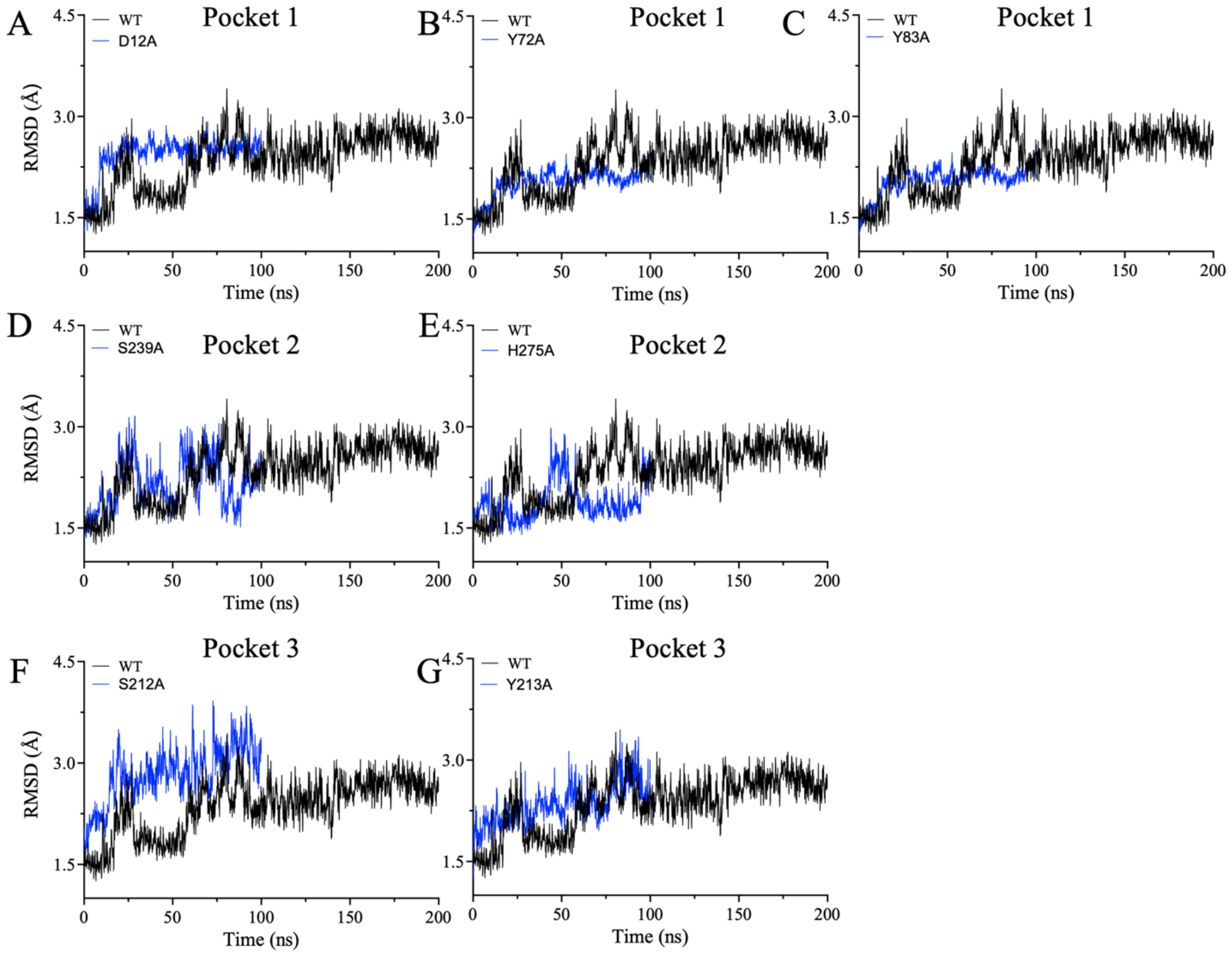

Figure S9: Molecular dynamics simulations of mutants in Pocket 1 – 3. The RMSD analysis of the protein backbone was conducted for both the WT and mutant PLpro structures in pockets 1 – 3 for 200 ns. (**A**–**C**) RMSD plots of mutants in Pocket 1: D12A, Y72A, and Y83A. (**D**, **E**) RMSD plots of mutants in Pocket 2: H275A and S239A. (**F**, **G**) RMSD plots of mutants in Pocket 3: S212A and Y213A. Minor differences between the alanine mutants and the WT PLpro enzyme have been observed.

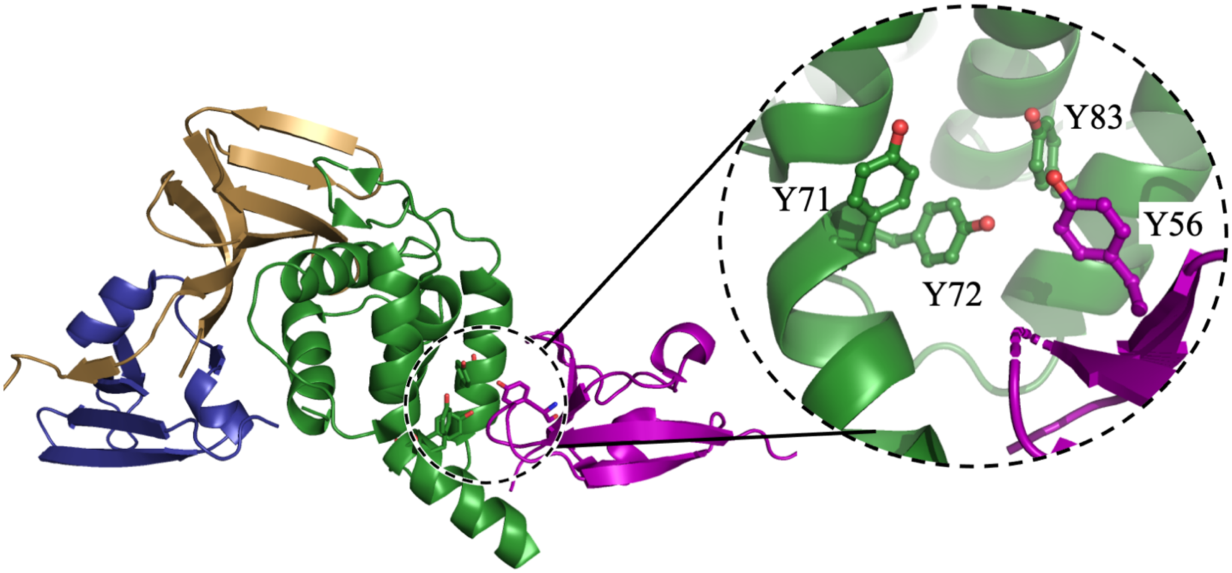

Figure S10: Cartoon representation of the interface interactions between the Ubl and thumb domains. Y56 (purple) of the Ubl domain forms a cluster with Y71, Y72, and Y83 (green) of the thumb domain. The tyrosine cluster is thought to stabilize the Ubl–thumb domain interface, which provides structural support for active PLpro. The figure was produced using PyMOL Molecular Graphics System version 2.5.5 (Schrödinger LLC).
